# Reappraising the role of instrumental inequalities for mendelian randomization studies in the mega Biobank era

**DOI:** 10.1007/s10654-023-01035-y

**Published:** 2023-08-27

**Authors:** Eleanor Sanderson, George Davey Smith

**Affiliations:** 1 MRC Integrative Epidemiology Unit, University of Bristol, Bristol, UK; 2 Population Health Sciences, Bristol Medical School, University of Bristol, Bristol, UK

Mendelian randomization (MR) uses the special properties of germline genetic variation to strengthen causal inference regarding modifiable exposures and health outcomes [1]. MR is now generally implemented within an instrumental variable (IV) framework. MR therefore depends on the IV assumptions being satisfied for the results obtained to be valid tests of the presence of a causal effect and estimators of the size of that causal effect. Two of these assumptions, that there is no confounding of the instrument and the outcome and that the instrument doesn’t affect the outcome other than through the exposure, cannot be show to be true and can only be falsified [2]. Instrumental inequalities are a set of inequality conditions that necessarily must hold if the IV assumptions hold. These therefore can be used to show when those conditions fail to hold, and so the instruments proposed are not valid instrumental variables [3, 4]. Showing that these inequalities are not satisfied within the data being analysed for an MR study can therefore indicate that the IV assumptions are not satisfied for that study. However, it is possible, or even likely, that the inequalities will be satisfied even if the proposed instruments violate the IV assumptions, therefore showing that the inequalities hold is not in itself particularly useful. Indeed instrumental inequalities were introduced in the early days of Mendelian randomization [3], but it was concluded at that time they were unlikely to be useful, given statistical power issues. Now we are in the era of mega Biobanks, however, the situation certainly deserves reappraisal.

In their paper Guo and colleagues set out to show whether the instrumental inequalities can detect violations of the IV assumptions in the estimation of six different exposures on coronary artery disease [5]. Through this they aim to illustrate the use and usefulness of instrumental inequalities to detect violations of the IV assumption in MR studies. They show that for the six exposures chosen (Vitamin D, Alcohol consumption, C-reactive protein, Triglycerides, HDL-Cholesterol and LDL-Cholesterol) all except Vitamin D violate the instrumental inequalities for the allele score. However, none of the individual genetic variants used for any of the exposures violate the IV inequalities.

Instrumental inequalities are applied to individual level data. Recently much development of methods for assumption testing in MR has focused on summary-data methods and there are a limited number of methods that can be used to assess the second and third IV assumptions with individual level data. Additionally many of the methods that do exist to assess the second and third assumption with individual level data rely on the genetic variants being included as individual instruments, and not combined in a score, which increases the potential for bias from many weak instruments in the analysis [6]. Instrumental inequalities can be applied to allele scores as well as individual genetic variants and therefore potentially provides a useful test in a setting where few are available.

As with all methods, there are limitations to the approach which it is important to be aware of. Instrumental inequalities require categorial exposures to be implemented, however all of the exposures, other than alcohol consumption, used by Guo et al. are continuous and so were categorized into deciles. Alcohol consumption was self-reported consumption categorized by the respondent based on frequency of consumption. Categorization of a truly continuous exposure into categories can lead to violations of the IV assumptions if the categorization is done inappropriately [7]. It is therefore possible that the results obtained were due to categorization cut offs chosen, rather than violation of the IV assumptions for the underlying continuous variable. For those variables where the researchers had control over the cut offs at the time of analysis (i.e. all other than alcohol consumption) it would be interesting to see how the choice of cut-offs varies the results obtained. The use of alternative exposures that are categorial in nature, such as educational attainment, may have been more informative about the application of instrumental inequalities.

An alternative approach that could be used as a test for whether the IV assumptions hold is to consider variance inflation of the outcome across the range of values of the instrument. If the IV assumptions hold then, conditional on the exposure, the variance of the outcome should be constant across different values of the instrument. Deviations from a constant variance would indicate potential violations of the IV assumptions [2]. Such an approach has been proposed elsewhere for detecting heterogeneous treatment effects in RCT’s [8]. Variance inflation can be applied to truly continuous exposures and so would overcome the issue of categorising an otherwise continuous trait.

A second limitation of this approach is the lack of identification of the individual SNPs that are causing the violation of the inequalities. As mentioned above, although all but one of the scores tested in this paper violated the IV assumptions, none of the individual SNPs did. The lack of any of the individual SNPs being identified is perhaps unsurprising as it has previously been suggested that with dichotomous instruments only extreme violations of the IV assumptions can be identified using instrumental inequalities [3]. As individual SNPs can only take three values (0/1/2 minor alleles) they are much closer to dichotomous instruments than an allele score. Individual SNPs are also more likely to be only weakly associated with the exposure, each individually violating the first IV assumption. The strength of the association between the SNPs and the exposure can be tested, but how this affects the ability of the instrumental inequalities to detect violations of the other IV assumptions is unclear.

The inability to identify which SNP(s) are most likely to be violating the IV assumptions highlights one limitation of the approach, without identifying which SNPs violate the IV assumptions it is not possible for the researcher to usefully act on the information provided by the instrumental inequalities test. They would therefore need to use alternative approaches which can be applied to individual level data, such as lasso selection [9] or the application of summary data methods to SNP-exposure and SNP-outcome associations generated from the data [10] to obtain results that were robust to those violations.

The only comparisons provided in the paper are to the MR-Egger intercept test [11] and MR-PRESSO global test [12]. The MR PRESSO global test also suggests that only Vitamin D satisfies the IV assumptions. The MR Egger intercept test also fails to reject for two other exposures (Alcohol consumption and LDL-Cholesterol), however MR Egger has low power to detect violations of the IV assumption and so a failure to reject is not strong evidence in support of the assumptions being satisfied in this case. A simple alternative test is the heterogeneity Q-statistic based on summary-statistics generated for each SNP [13, 14]. An advantage of the Q-statistic is that the individual contribution of each SNP to the overall test can be calculated. It would be interesting to see instrumental inequalities compared to other approaches to detect violations of the IV assumptions, and whether the approach of excluding SNPs to reduce the total Q-statistic would also lead to the equivalent score satisfying the instrumental inequalities conditions. Considerable further work along these and other lines is necessary before the value of instrumental inequalities in Mendelian randomization studies becomes clear.
